# Precision and Agreement of Corneal Power Measurements Obtained Using a New Corneal Topographer OphthaTOP

**DOI:** 10.1371/journal.pone.0109414

**Published:** 2015-01-05

**Authors:** Jinhai Huang, Giacomo Savini, Hao Chen, Fangjun Bao, Yuanguang Li, Haisi Chen, Weicong Lu, Ye Yu, Qinmei Wang

**Affiliations:** 1 School of Optometry and Ophthalmology and Eye Hospital, Wenzhou Medical University, Wenzhou, Zhejiang, China; 2 Key Laboratory of Vision Science, Ministry of Health P.R. China, Wenzhou, Zhejiang, China; 3 Studio Oculistico d’Azeglio, Bologna, Italy; Bascom Palmer Eye Institute, University of Miami School of Medicine, United States of America

## Abstract

**Purpose:**

To evaluate repeatability and reproducibility of anterior corneal power measurements obtained with a new corneal topographer OphthaTOP (Hummel AG, Germany) and agreement with measurements by a rotating Scheimpflug camera (Pentacam HR, Oculus, Germany) and an automated keratometer (IOLMaster, Carl Zeiss Meditec, Germany).

**Methods:**

The right eyes of 79 healthy subjects were prospectively measured three times with all three devices. Another examiner performed three additional scans with the OphthaTOP in the same session. Within one week, the first examiner repeated the measurements using the OphthaTOP. The flat simulated keratometry (Kf), steep K (Ks), mean K (Km), J_0_, and J_45_ were noted. Repeatability and reproducibility of measurements were assessed by within-subject standard deviation (Sw), repeatability (2.77 Sw), coefficient of variation (CoV), and intraclass correlation coefficient (ICC). Agreement between devices was assessed using 95% limits of agreement (LoA).

**Results:**

Intraobserver repeatability and interobserver and intersession reproducibility of all measured parameters showed a 2.77 Sw of 0.29 diopter or less, a CoV of less than 0.24%, and an ICC of more than 0.906. Statistically significant differences (*P*<0.001) were found between the parameters analyzed by the three devices, except J_0_ and J_45_. The mean differences between OphthaTOP and the other two devices were small, and the 95% LoA was narrow for all results.

**Conclusions:**

The OphthaTOP showed excellent intraobserver repeatability and interobserver and intersession reproducibility of corneal power measurements. Good agreements with the other two devices in these parameters were found in healthy eyes.

## Introduction

The requirement for precise measurements and assessments of corneal topography is increasing. Most patients undergoing cataract or refractive surgery expect to experience excellent postoperative uncorrected visual acuity, [1] and for this purpose an accurate and precise examination of such patients’ ocular parameters is mandatory.[2]–[6] In addition, corneal power is indispensable to analyzing the shape of the cornea and for fitting contact lenses.[7]–[10] Incorrect or invalid information may result in misdiagnosing the keratoconus or misjudging the appropriate timing of treatment. And it will lead to errors in contact lens design and fitting and in the visual outcome, increasing discomfort and complications.

At present, several types of commercially available technologies can be used to measure the corneal curvature and calculate the corneal power.[2], [11] Automated keratometry (e.g., IOLMaster, Carl Zeiss Meditec, Germany)[12], [13] measures the cornea curvature by analyzing the distance between the reflected images. Videokeratography systems perform corneal topography by reflecting Placido disk rings on the cornea and then extracting curvature data from these images of the anterior corneal surface.[14], [15] Corneal tomography, such as provided by rotating Scheimpflug cameras, can provide a relatively whole image of the anterior segment of the eye to analyze corneal power and other parameters. Examples of this technology include Sirius (CSO, Italy); Galilei (Ziemer, Switzerland); Pentacam (Oculus Optikgeräte GmbH, Germany); and TMS-5 (Tomey, Japan) [5], [16].

The OphthaTOP (Hummel AG, Germany) is a new Placido disk-based corneal topographer. To our knowledge, the precision of this device in corneal power measurement had never been studied. In addition, there were no studies that represented the agreement between this new device and other instruments, such as Pentacam or IOLMaster, which are widely used in common clinical practice.

The purpose of this study was to prospectively evaluate the intraoperator repeatability, interoperator and intersession reproducibility of corneal power measurements derived by the OphthaTOP, and then to estimate agreement of these measurements with those obtained using the Pentacam and IOLMaster.

## Subjects and Methods

### Ethics Statement

The study was conducted at the Eye Hospital of Wenzhou Medical University. The research was performed in accordance with the principles stated in the Declaration of Helsinki and was approved by the Office of Research Ethical Committee, Wenzhou Medical University. All participants provided written informed consent after the nature of the study had been explained to them.

### Subjects

This prospective study enrolled 79 normal, healthy subjects, including 26 males and 53 females. Only the right eye of each subject was selected for measurement. The mean age was 25.22±6.99 years (range 22 to 62 years), and the mean spherical equivalent refraction was −3.58±2.35 diopters (range 0 to −9.5 diopters). Inclusion criteria were healthy subjects without communication or cooperation disorders. All the subjects had good fixation ability, and their intraocular pressures were in the range of 10 mmHg to 21 mmHg. The exclusion criteria were 1) history of ocular pathological changes; 2) history of corneal or intraocular trauma; 3) previous ophthalmologic operation; 4) wearing soft contact lens within 2 weeks or rigid gas permeable contact lenses within 4 weeks; 5) dry eye (significant subjective symptoms, or tear film break-up time shorter than 5 seconds); and 6) acute or chronic systemic severe disease.

### Instruments

The OphthaTOP is a new device based on the Placido disk to get corneal topography. In the present study, software version V2.19 was used. It projects 30 rings onto the cornea and acquires the data of 10,800 measurement points at a time. All images are captured automatically once the green spot for starting, and the red spot for laser locating, are adjusted at the appropriate positions. During each scan, the device captures seven images continually, and then combines these images to get a final corneal topography. It converts the curvature results of the anterior corneal surface into a cornea dioptric value using the formula below:
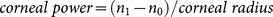
where n_1_ is the keratometric index (1.3375) and n_0_ represents the refractive index of air (1.0000). In this study, we analyzed the two perpendicular meridians with the largest curvature difference within a diameter of about 3.5 mm to get the keratometry (K) value.

The Pentacam HR uses a rotating Scheimpflug camera to get the image of the anterior segment of the eye. It captures 138,000 true elevation points using a high-resolution, 1.45 mega-pixel camera. In the present study, the automatic release mode was used and 25 slit images of the anterior segment were obtained in less than 2 seconds. Only the results with an examination quality specification of “OK” were accepted for analysis. A keratometric index of 1.3375 was also used for calculation of corneal power and the K values were obtained via the data from a diameter of about 3.0 mm.

The IOLMaster works according to the optical principle of reflection by the anterior surface of six bright spots in the center of the cornea. The six points are reflected at a diameter of approximately 2.5 mm. The keratometric index of 1.3375 was used to calculate the corneal power.

### Measurement Protocol

The present study’s definitions of repeatability, reproducibility, and agreement were based on those adopted by the British Standards Institute and the International Organization for Standardization.[17]–[19] Calibration of each device was performed by the manufacturer prior to data collection. The sequence of the examiners was in consecutive order. Each subject was measured by the first experienced examiner using all three devices to determine intraobserver repeatability, and each device performed three times with valid results. Three additional consecutive scans were obtained by a second experienced observer using the new corneal topographer to determine interoperator reproducibility. Then the measurements were repeated by the first observer within 2 days to 7 days to determine intersession reproducibility. The second session was carried out at almost the same time as the first session. The sequence of the devices was randomly chosen. Less than 5 minutes were spent when each subject was examined with the OphthaTOP and the total time of all devices measurements was less than 30 minutes. All subjects were asked to perform a complete blink every time just before measurement, and they were told to sit back after each measurement to ensure the device was realigned before the next measurement. All the measurements were carried out at least 3 hours after the subjects woke from sleep. Examinations were taken at 10 AM and 5 PM. And all subjects were affirmed to have avoided substantial reading prior to the measurements [20].

### Statistical Analysis

All data were analyzed using SPSS software for Windows version 20 (SPSS Inc., Chicago, USA) and MedCalc Statistical Software version 13.0 (MedCalc Software, Inc., Mariakerke, Belgium). A *P* value less than 0.05 was considered to be statistically significant. The distribution of all the data obtained was not significantly different from normal after checking with Kolmogorov-Smirnov tests (*P*>0.05). During each measurement, the flat (Kf) and steep keratometry (Ks) values, the average keratometry (Km), and the axes of Kf and Ks were acquired. Corneal astigmatism was converted into a vector representation of Jackson J_0_ and J_45_. J_0_ means cylinder at 0-degree meridian, and J_45_ means cylinder at 45-degree meridian. They were calculated according to the following formulas [21]:
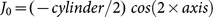


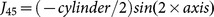



Where the cylinder was the corneal astigmatism magnitude, i.e. the difference between Ks and Kf, and the axis was the meridian of Ks.

These values were calculated for three measurements during each session and then averaged to determine the reproducibility and comparability.

To determine the intrasession repeatability of OphthaTOP, the within-subject standard deviation (Sw), test-retest repeatability (TRT), within-subject coefficient of variation (CoV), and intraclass correlation coefficients (ICCs) were calculated for the three repeated measurements obtained by the two examiners. The test-retest repeatability was defined as 2.77 Sw, which means an interval within which 95% of the differences between measurements are expected to lie.[22] The CoV was calculated as the ratio of the Sw to the overall mean (a lower CoV stands for higher repeatability). Using the CoV values, data with different units or widely different means can be compared between each other. However, the disadvantage is that when the mean value is near zero, the CoV becomes sensitive to small changes in the mean. Because the mean values of J_0_ and J_45_ are both near zero, we did not calculate the CoV for them. The ICCs (ranging from 0 to 1) are a reliability coefficient calculated from variance estimates obtained through an analysis of variance, and a value more than 0.9 indicates acceptable clinical reliability.[23], [24] To assess interoperator and intersession reproducibility, the mean of the three readings from each operator and session was calculated for each device first. Then the interoperator and intersession Sw, 2.77 Sw, CoV, and ICCs were calculated.

Repeated-measures analysis of variance (ANOVA) with the Bonferroni correction was used to identify pairs that were significantly different. Agreement among the devices was assessed according to the method described by Bland and Altman. It involved the use of the 95% limits of agreement (LoA) as the mean difference ±1.96 standard deviation (SD). Narrower 95% LoA is associated with higher agreement between each other [19].

## Results

### Repeatability and reproducibility of corneal power measurements obtained using the OphthaTOP corneal topographer

Table 1 shows the mean values Sw, 2.77 Sw, CoV, and ICCs for the Kf, Ks, Km, and power vectors J_0_ and J_45_ for the three continuous measurements. The CoV values of K were no more than 0.24%, and the ICCs were more than 0.9 for all parameters. Therefore, this new device shows high intraobserver repeatability in measuring corneal power.

**Table 1:** Intraobserver Repeatability of Flat Keratometry (Kf), Steep Keratometry (Ks), Mean Keratometry (Km), Vector J_0_ and J_45_ Measurements Obtained Using the OphthaTOP Conceal Topographer.

Parameters	observer	Mean (D) ± SD	Sw (D)	2.77 Sw (D)	CoV (%)	ICC
Kf	1st	42.87±1.33	0.09	0.24	0.20	0.996
	2nd	42.85±1.33	0.10	0.27	0.23	0.994
Ks	1st	43.93±1.50	0.10	0.29	0.24	0.995
	2nd	43.95±1.51	0.10	0.28	0.23	0.996
Km	1st	43.41±1.40	0.08	0.21	0.18	0.997
	2nd	43.39±1.39	0.09	0.24	0.20	0.996
J_0_	1st	−0.49±0.33	0.06	0.17		0.966
	2nd	−0.49±0.32	0.05	0.14		0.975
J_45_	1st	0.00±0.15	0.05	0.13		0.906
	2nd	0.00±0.15	0.04	0.11		0.930

D = diopter, SD = standard deviation, Sw = within-subject standard deviation, CoV = within-subject coefficient of variation, ICC = intraclass correlation coefficient.

Table 2 shows the mean values Sw, 2.77 Sw, CoV, and ICCs of the Kf, Ks, Km, J_0_, and J_45_ for the assessment of interobserver reproducibility. The CoV values of K were less than 0.20%, the Sw and 2.77 Sw values were within 0.09D and 0.24D. The Sw and 2.77 Sw of J_0_ and J_45_ values were within 0.04D and 0.11D, respectively, and ICCs of all parameters were above 0.94. The results indicate that the interobserver reproducibility of this new topographer was excellent.

**Table 2:** Interobserver reproducibility of Flat Keratometry (Kf), Steep Keratometry (Ks), Mean Keratometry (Km), Vector J_0_ and J_45_ Measurements Obtained Using the OphthaTOP Conceal Topographer.

Parameter	Mean (D) ± SD	Sw (D)	2.77 Sw (D)	CoV (%)	ICC
Kf	42.86±1.33	0.06	0.17	0.14	0.998
Ks	43.94±1.50	0.09	0.24	0.20	0.997
Km	43.40±1.39	0.06	0.17	0.14	0.998
J_0_	−0.49±0.32	0.04	0.11		0.984
J_45_	0.00±0.14	0.04	0.10		0.942

D = diopter, SD = standard deviation, Sw = within-subject standard deviation, CoV = within-subject coefficient of variation, ICC = intraclass correlation coefficient.

There were also no significant differences in the measurements between the first and the second session (Table 3). The Sw and 2.77 Sw values of Kf, Ks, and Km were within 0.10D and 0.29D. The CoV values were less than 0.24%. The Sw and 2.77 Sw of J_0_ and J_45_ values were within 0.05D and 0.13D, respectively.

**Table 3:** Intersession reproducibility of Flat Keratometry (Kf), Steep Keratometry (Ks), Mean Keratometry (Km), Vector J_0_ and J_45_ Measurements Obtained Using the OphthaTOP Conceal Topographer.

Parameter	Mean (D) ± SD	Sw (D)	2.77 Sw (D)	CoV (%)	ICC
Kf	42.87±1.34	0.08	0.21	0.18	0.997
Ks	43.94±1.52	0.10	0.29	0.24	0.995
Km	43.41±1.41	0.08	0.22	0.18	0.997
J_0_	−0.48±0.33	0.05	0.13		0.980
J_45_	0.00±0.14	0.04	0.11		0.921

D = diopter, SD = standard deviation, Sw = within-subject standard deviation, CoV = within-subject coefficient of variation, ICC = intraclass correlation coefficient.

### Comparison of devices

**Figure 1:**
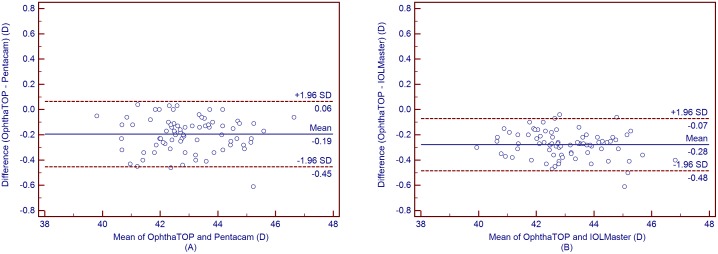
Bland-Altman plots showing the means plotted against the differences in values of flat K for a comparison between the new corneal topographer OphthalTop and Pentacam Scheimpflug camera (A) and between the new corneal topographer and IOLMaster automated keratometer (B). The solid line indicates the mean difference (bias). The upper and lower lines represent the 95% LoA.

**Figure 2:**
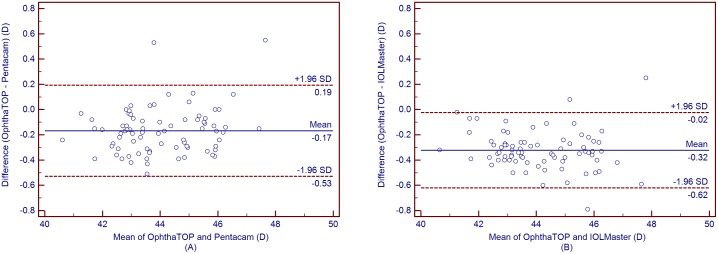
Bland-Altman plots showing the means plotted against the differences in values of steep K for a comparison between the new corneal topographer OphthalTop and Pentacam Scheimpflug camera (A) and between the new corneal topographer and IOLMaster automated keratometer (B). The solid line indicates the mean difference (bias). The upper and lower lines represent the 95% LoA.

**Figure 3:**
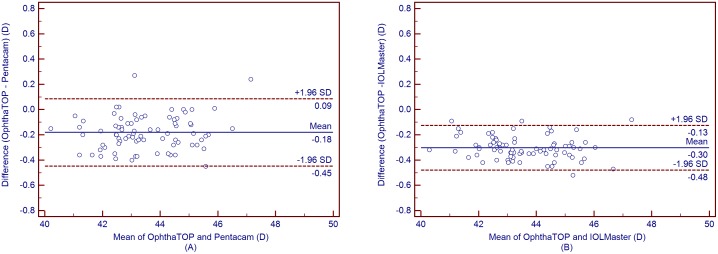
Bland-Altman plots showing the means plotted against the differences in values of mean K for a comparison between the new corneal topographer OphthalTop and Pentacam Scheimpflug camera (A) and between the new corneal topographer and IOLMaster automated keratometer (B). The solid line indicates the mean difference (bias). The upper and lower lines represent the 95% LoA.

**Figure 4:**
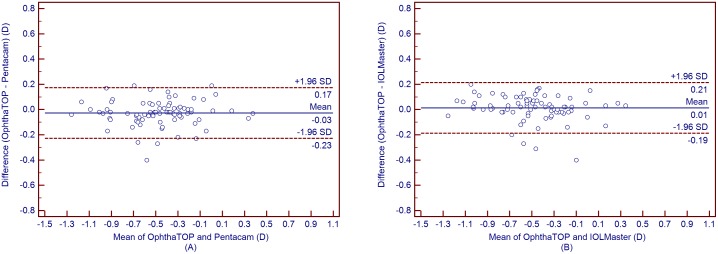
Bland-Altman plots showing the means plotted against the differences in values of vector J_0_ for a comparison between the new corneal topographer OphthalTop and Pentacam Scheimpflug camera (A) and between the new corneal topographer and IOLMaster automated keratometer (B). The solid line indicates the mean difference (bias). The upper and lower lines represent the 95% LoA.

**Figure 5:**
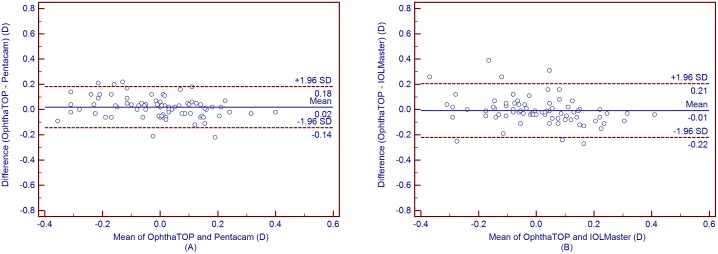
Bland-Altman plots showing the means plotted against the differences in values of vector J_45_ for a comparison between the new corneal topographer OphthalTop and Pentacam Scheimpflug camera (A) and between the new corneal topographer and IOLMaster automated keratometer (B). The solid line indicates the mean difference (bias). The upper and lower lines represent the 95% LoA.

Table 4 shows the mean corneal power measurements by the Pentacam and IOLMaster. The Kf, Ks, and Km values obtained using the OphthaTOP and the Pentacam were statistically different (*P*<0.001), while the J_0_ and J_45_ values showed no statistical difference (*P*>0.05) (Table 5). Furthermore, a similar result was found between the parameters measured by the OphthaTOP and the IOLMaster, as the Kf, Ks, and Km values were statistically different (*P*<0.001), whereas J_0_ and J_45_ did not show any statistically significant difference (*P*>0.05) (Table 6). Although both Pentacam and IOLMaster showed higher corneal power readings than the OphthaTOP, the 95% LoA was narrow–i.e. close or lower than 0.50D in all cases. This means that agreement among these devices was relatively good (Figs. 1 to 5).

**Table 4:** Flat Keratometry (Kf), Steep Keratometry (Ks), Mean Keratometry (Km), Vector J0 and J45 Measurements Obtained Using the Pentacam Rotating Scheimpflug System and the IOLMaster.

Parameter	Mean (D) ± SD	
	Pentacam	IOLMaster
Kf	43.06±1.34	43.15±1.35
Ks	44.12±1.47	44.27±1.53
Km	43.59±1.38	43.71±1.42
J_0_	−0.46±0.33	−0.50±0.35
J_45_	−0.02±0.17	0.01±0.18

D = diopter, SD = standard deviation.

**Table 5:** Comparison of Flat Keratometry (Kf), Steep Keratometry (Ks), Mean Keratometry (Km), Vector J_0_ and J_45_ Measurements Obtained Using the OphthaTOP Conceal Topographer and the Pentacam Rotating Scheimpflug System.

Parameter	Mean Difference ± SD	*P* Value	95% LoA
Kf (D)	−0.19±0.13	<0.001	−0.45 to 0.06
Ks (D)	−0.17±0.18	<0.001	−0.53 to 0.19
Km (D)	−0.18±0.14	<0.001	−0.45 to 0.09
J_0_	−0.03±0.10	0.059	−0.23 to 0.17
J_45_	0.02±0.08	0.133	−0.14 to 0.18

D = diopter, SD = standard deviation, LoA = limits of agreement.

**Table 6:** Comparison of Flat Keratometry (Kf), Steep Keratometry (Ks), Mean Keratometry (Km), Vector J_0_ and J_45_ Measurements Obtained Using the OphthaTOP Conceal Topographer and the IOLMaster.

Parameter	Mean Difference ± SD	*P* Value	95% LoA
Kf (D)	−0.28±0.11	<0.001	−0.48 to −0.07
Ks (D)	−0.32±0.15	<0.001	−0.62 to −0.02
Km (D)	−0.30±0.09	<0.001	−0.48 to −0.13
J_0_	0.01±0.10	0.772	−0.19 to 0.21
J_45_	−0.01±0.11	1.000	−0.22 to 0.21

D = diopter, SD = standard deviation, LoA = limits of agreement.

## Discussion

More and more corneal topographers are being produced and applied to clinical routine. This study was aimed at evaluating the precision and agreement of a new corneal topographer, the OphthaTOP. Our data showed high intraobserver repeatability of the Placido disk-based corneal topographer’s measurement of corneal power, with a low Sw (no more than 0.10D), a low CoV (less than 0.25%), and high ICC (more than 0.90) values.

The current results are comparable to the repeatability of other Placido disk-based corneal topographers. The Medmont E300 (Medmont Pty Ltd, Melbourne, Australia) is a Placido disk-based videokeratoscope. It has 32 Placido rings, measures 9,600 data points during every scan, and has been found to be highly accurate and repeatable.[9], [25] Chui et al.[26] found the repeatability of the Medmont was good for the measurement of apical radius (R_0_) and flattest corneal curvature, and the 95% LoA values of R_0_ and flattest corneal curvature between the two measurements were −0.06 to +0.06 mm and −0.10 to +0.13 mm, respectively.

Another instrument–the EyeSys System (EyeSys Vision, INC, Houston, Texas, USA), which contains 26 Placido disks and measures 9,360 points–had been reported for its high repeatability in corneal curvature measurement by Gonzalez Perez et al.[27] and Jeandervin et al.[28] Both studies were conducted using calibration surface, and the coefficient of repeatability was less than 0.06 mm for all examiners in the former. Wang et al.[29] compared eight different devices in their study and found high repeatability of Medmont, with a small 2.77 Sw and CoV values of less than 0.23D and 0.18%, respectively, and high ICCs (>0.997). The 2.77 Sw, CoV, and ICC values of EyeSys were less than 0.36D, less than 0.30%, and more than 0.989, respectively. Those values with the Topolyzer (WaveLight Technologie AG, Erlangen, Germany), a videokeratoscope that contains 22 Placido rings and analyzes 22,000 data points, were less than 0.35D, less than 0.29%, and above 0.993, respectively. Compared with the EyeSys and Topolyzer, the 2.77 Sw and CoV values of the OphthaTOP in our study were even lower.

In addition, we got similarly high interobserver reproducibility and intersession reproducibility of corneal power values captured by the OphthaTOP. The maximum 2.77 Sw, maximum CoV, and minimum ICCs values were 0.24D, 0.20%, 0.942 and 0.29D, 0.24%, 0.921, respectively. Compared with the Keratron (Optikon 2000 SpA, Rome, Italy)–which was found to have good interobserver reproducibility without statistically significant difference of the mean values between two observers and with a 95% LoA for the flattest meridian (ranging from −0.50 to +0.33D) and the steepest meridian (ranging from −0.53 to +0.31D)[30]–the OphthaTOP seems to perform better in the current study.

The measurements with OphthaTOP were comparable to the study of Mao et al.[31], in which the Placido disk-based corneal topographer Keratograph 4 (Oculus Optikgeräte GmbH, Germany) had shown excellent interoperator reproducibility and intersession reproducibility. The CoV of K values were within 0.15% and 0.21%, respectively, and the Sw and 2.77 Sw of all parameters were within 0.06D, 0.17D, and 0.09D, 0.25D, respectively.

To our knowledge, no previous study had tested agreement of the OphthaTOP with other devices in corneal power measurement. Previous studies had shown that the Pentacam and IOLMaster, which are both widely used and validated devices, have good precision and agreement.[12], [29], [32]–[34] Therefore, we compared the OphthaTOP with these two instruments, and our results confirmed relatively good agreement between the OphthaTOP and Pentacam and between the OphthaTOP and IOLMaster, although the OphthaTOP provided lower mean corneal powers.

As a representative of the rotating Scheimpflug camera system, Pentacam had shown its positive repeatability and reproducibility in quite a few studies previously.[32], [34] In earlier studies, it had been compared with other instruments that are based on the Placido disk. Kawamorita et al.[30] found that mean axial power for the central corneal power values obtained by Pentacam was statistically significantly lower than the Keratron (P<0.003), and the 95% LoA values between the two devices for the Kf and Ks were −1.16 to +0.28D and −1.06 to +0.55D, respectively. In the study by Savini et al., [35] the 95% LoA of the Km values between Pentacam and TMS-2 (Tomey, Erlangen, Germany) and between the Pentacam and Keratron reached −1.05 to +0.94D and −0.95 to +1.02D, respectively. However, Scott et al.[36] found good agreement between the Pentacam and the Medmont for measurements of axial power best-fit spherocylinder power vectors; the maximum range of the LoA values were 0.40D to −0.17D. With the data of the current study, the mean values of the corneal power were close between the devices and the 95% LoA of all parameters were narrow, with the maximum 95% LoA range of −0.53 to 0.19D.

In a previous study of IOLMaster, the IOLMaster provided statistically significantly steeper readings than the RK-F1 AutoRef-Keratometer (Canon Inc., Japan) (P<0.001), with the mean difference of the Kf and Ks within 0.29D (the range of 95% LoA was −0.08 to 0.66D) and 0.18D (the range of 95% LoA was −0.34 to 0.66D).[37] When compared with the manual keratometry, the IOLMaster showed higher agreement than some other devices, such as EyeSys. The mean difference of minimum keratometry, maximum keratometry, and mean keratometry between the manual Javal keratometer (Haag-Streit) and IOLMaster was just 0.09±0.24D, 0.27±0.41D, and 0.18±0.24D, respectively, and the 95% LoA was −0.57D to 0.38D, −1.06 to 0.53D, and −0.66 to 0.30D, respectively.[11] In our study, the IOLMaster produced the highest corneal power values among all the devices. The mean difference between the OphthaTOP and IOLMaster was −0.32±0.15D, and the 95% LoA values were in a narrow range of −0.62 to −0.02D. Some other studies comparing the IOLMaster with other corneal topographers found that it produces higher readings, which is in agreement with our results. Wang et al.[29] found a maximum mean difference of 0.13±0.21D, with a maximum 95% LoA range of −0.68 to 0.06D between the IOLMaster and the Topolyzer. Mao et al.[31] reported a maximum 95% LoA range of −0.28 to 0.62D between the IOLMaster and Keratograph 4, with relatively higher values generated by the former. In the present study, the new corneal topographer provided the flattest corneal power values. As is well known, the shape of the corneal surface is not a sphere but rather can be described through a conic section by the radius of curvature and asphericity measurement.[38]–[40] The corneal curvature values are steeper in the central area and become flatter in the peripheral zones. Among these instruments, the analyzing area of the OphthaTOP is the largest, with a diameter of 3.5 mm, while the area of Pentacam is 3.0 mm and the IOLMaster is just 2.5 mm. This, to some extent, could explain the difference of corneal power values among the devices.

With regard to the analysis of astigmatism, we converted the keratometric values into the Jackson cross cylinder notation J_0_ and J_45_, as previously described by Thibos et al. [21] Good agreement among different devices in measuring corneal astigmatism has not been found by all studies. When assessing agreement of corneal astigmatism between Placido topography and Scheimpflug tomography, Delrivo et al. [41] found a statistically significant difference in the J_0_ and not in the J_45_ vector. Greater differences were observed in lower degrees of astigmatism. However, in the present study, neither J_0_ nor J_45_ showed any statistically significant difference between the devices, although the astigmatic magnitude was always lower than 2 D. Similar results were shown in the Mao et al.’s [31] study, as no clinically significant difference was found between the Keratograph 4 and IOLMaster or Pentacam.

The performance of videokeratoscopes is limited by several other factors, including alignment and focusing techniques, camera resolution, and the computer algorithms of the instrument.[25] The OphthaTOP is designed to capture images automatically when the two dots are aligned in a small area, and good alignment and focusing techniques are also ensured in the other two devices. Muller et al.[42] found that the rigidity of the most anterior part of the corneal stroma in extreme hydration states plays an important role in maintenance of corneal curvature. Swelling of the surface of the cornea will increase variance, so we conducted our examination after the subjects had been awake for hours.

This study has some limitations, warranting further investigation. The age distribution of subjects we enrolled was not very wide. The understanding, collaboration, and fixation of all the subjects were good, but according to our experience, the subjects had to keep their eyes open as widely as possible to ensure a perfect image capture using the new corneal topographer, so additional studies are needed to evaluate the precision and agreement in older subjects and in children. Tang et al.[25] chose six different test surfaces to evaluate the reliability of different topographers, but the performance of the devices was just good on certain surfaces. In our study, the subjects were mostly normal, healthy individuals without too much shape variation, and the degree of astigmatism was no more than −2.0 diopter, so further studies are needed to determine the performance in patients with high astigmatism or keratoconus. Furthermore, other Placido-based devices analyzing corneal topography are commercially available, and more studies should be carried out to assess their agreement. Finally, another major function of the OphthaTOP is intraocular lens power calculation, and additional investigation could be made to assess achievement of this purpose.

In summary, the corneal power values provided by the OphthaTOP corneal topographer showed excellent intraobserver repeatability and interobserver and intersession reproducibility in corneal power measurement of normal healthy eyes. Our data showed good agreement between the OphthaTOP and Pentacam and between the OphthaTOP and IOLMaster, notwithstanding a statistically significant difference in corneal power measurements, whose mean values were lower with the OphthaTOP.

## Supporting Information

S1 File Corneal power data file.
(XLS)

## References

[1] Parede TR, Torricelli AA, Mukai A, Vieira Netto M, Bechara SJ (2013) Quality of vision in refractive and cataract surgery, indirect measurers: review article. Arq Bras Oftalmol 76:386–390. 24510091 10.1590/s0004-27492013000600016

[2] Tang M, Li Y, Avila M, Huang D (2006) Measuring total corneal power before and after laser in situ keratomileusis with high-speed optical coherence tomography. J Cataract Refract Surg 32:1843–1850. 17081867 10.1016/j.jcrs.2006.04.046 PMC1808223

[3] Ladas JG, Boxer Wachler BS, Hunkeler JD, Durrie DS (2001) Intraocular lens power calculations using corneal topography after photorefractive keratectomy. Am J Ophthalmol 132:254–255. 11476688 10.1016/s0002-9394(01)00894-7

[4] Aristodemou P, Knox Cartwright NE, Sparrow JM, Johnston RL (2011) Formula choice: Hoffer Q, Holladay 1, or SRK/T and refractive outcomes in 8108 eyes after cataract surgery with biometry by partial coherence interferometry. J Cataract Refract Surg 37:63–71. 21183100 10.1016/j.jcrs.2010.07.032

[5] Rao SN, Raviv T, Majmudar PA, Epstein RJ (2002) Role of Orbscan II in screening keratoconus suspects before refractive corneal surgery. Ophthalmology 109:1642–1646. 12208710 10.1016/s0161-6420(02)01121-1

[6] Frings A, Katz T, Steinberg J, Druchkiv V, Richard G, et al (2014) Ocular residual astigmatism: Effects of demographic and ocular parameters in myopic laser in situ keratomileusis. J Cataract Refract Surg 40:232–238. 24333012 10.1016/j.jcrs.2013.11.015

[7] Lloyd McKernan A, O’Dwyer V, Simo Mannion L (2014) The influence of soft contact lens wear and two weeks cessation of lens wear on corneal curvature. Cont Lens Anterior Eye 37:31–37. 23973214 10.1016/j.clae.2013.07.014

[8] Kamiya K, Ishii R, Shimizu K, Igarashi A (2014) Evaluation of corneal elevation, pachymetry and keratometry in keratoconic eyes with respect to the stage of Amsler-Krumeich classification. Br J Ophthalmol. 10.1136/bjophthalmol-2013-304132 24457362

[9] Cho P, Lam AK, Mountford J, Ng L (2002) The performance of four different corneal topographers on normal human corneas and its impact on orthokeratology lens fitting. Optom Vis Sci 79:175–183. 11915858 10.1097/00006324-200203000-00012

[10] Liu Z, Xie Y, Zhang M (2001) Corneal topography and pachymetry in normal eyes. Zhonghua Yan Ke Za Zhi 37:125–128. 11864407

[11] Mehravaran S, Asgari S, Bigdeli S, Shahnazi A, Hashemi H (2014) Keratometry with five different techniques: a study of device repeatability and inter-device agreement. Int Ophthalmol. 10.1007/s10792-013-9895-3 24562593

[12] Santodomingo-Rubido J, Mallen EA, Gilmartin B, Wolffsohn JS (2002) A new non-contact optical device for ocular biometry. Br J Ophthalmol 86:458–462. 11914218 10.1136/bjo.86.4.458 PMC1771084

[13] Elbaz U, Barkana Y, Gerber Y, Avni I, Zadok D (2007) Comparison of different techniques of anterior chamber depth and keratometric measurements. Am J Ophthalmol 143:48–53. 17101110 10.1016/j.ajo.2006.08.031

[14] Moura RC, Bowyer BL, Stevens SX, Rowsey JJ (1998) Comparison of three computerized videokeratoscopy systems with keratometry. Cornea 17:522–528. 9756447 10.1097/00003226-199809000-00010

[15] Varssano D, Rapuano C, Luchs J (1997) Comparison of keratometric values of healthy and diseased eyes measured by Javal keratometer, EyeSys, and PAR. J Cataract Refract Surg 23:419–422. 9159687 10.1016/s0886-3350(97)80187-3

[16] Kawamorita T, Uozato H, Kamiya K, Bax L, Tsutsui K, et al (2009) Repeatability, reproducibility, and agreement characteristics of rotating Scheimpflug photography and scanning-slit corneal topography for corneal power measurement. J Cataract Refract Surg 35:127–133. 19101435 10.1016/j.jcrs.2008.10.019

[17] Institution. BS (1994) Accuracy (Trueness and Precision) of Measurement Methods and Results: General Principles and Definitions. London: HMO BS ISO 5725 part 1.

[18] Institution. BS (1994) Accuracy (Trueness and Precision) of Measurement Methods and Results: Basic Methods for the Determination of Repeatability and Reproducibility of a Standard Measurement Method. London: HMO BS ISO 5725 part 2.

[19] Bland JM, Altman DG (1986) Statistical methods for assessing agreement between two methods of clinical measurement. Lancet 1:307–310. 2868172

[20] Collins MJ, Buehren T, Bece A, Voetz SC (2006) Corneal optics after reading, microscopy and computer work. Acta Ophthalmol Scand 84:216–224. 16637840 10.1111/j.1600-0420.2005.00547.x

[21] Thibos LN, Wheeler W, Horner D (1997) Power vectors: an application of Fourier analysis to the description and statistical analysis of refractive error. Optom Vis Sci 74:367–375. 9255814 10.1097/00006324-199706000-00019

[22] Bland JM, Altman DG (1996) Measurement error. BMJ 313:744. 8819450 10.1136/bmj.313.7059.744 PMC2352101

[23] Muller R, Buttner P (1994) A critical discussion of intraclass correlation coefficients. Stat Med 13:2465–2476. 7701147 10.1002/sim.4780132310

[24] Kramer MS, Feinstein AR (1981) Clinical biostatistics. LIV. The biostatistics of concordance. Clin Pharmacol Ther 29:111–123. 7460469 10.1038/clpt.1981.18

[25] Tang W, Collins MJ, Carney L, Davis B (2000) The accuracy and precision performance of four videokeratoscopes in measuring test surfaces. Optom Vis Sci 77:483–491. 11014675 10.1097/00006324-200009000-00009

[26] Chui WS, Cho P (2005) A comparative study of the performance of different corneal topographers on children with respect to orthokeratology practice. Optom Vis Sci 82:420–427. 15894918 10.1097/01.OPX.0000162642.24885.71

[27] Gonzalez Perez J, Cervino A, Giraldez MJ, Parafita M, Yebra-Pimentel E (2004) Accuracy and precision of EyeSys and Orbscan systems on calibrated spherical test surfaces. Eye Contact Lens 30:74–78. 15260351 10.1097/01.icl.0000111749.04644.92

[28] Jeandervin M, Barr J (1998) Comparison of repeat videokeratography: repeatability and accuracy. Optom Vis Sci 75:663–669. 9778699 10.1097/00006324-199809000-00021

[29] Wang Q, Savini G, Hoffer KJ, Xu Z, Feng Y, et al (2012) A comprehensive assessment of the precision and agreement of anterior corneal power measurements obtained using 8 different devices. PLoS One 7:e45607. 23049823 10.1371/journal.pone.0045607 PMC3458095

[30] Kawamorita T, Nakayama N, Uozato H (2009) Repeatability and reproducibility of corneal curvature measurements using the Pentacam and Keratron topography systems. J Refract Surg 25:539–544. 19603622 10.3928/1081597X-20090512-08

[31] Mao X, Savini G, Zhuo Z, Feng Y, Zhang J, et al (2013) Repeatability, reproducibility, and agreement of corneal power measurements obtained with a new corneal topographer. J Cataract Refract Surg 39:1561–1569. 23860010 10.1016/j.jcrs.2013.04.029

[32] Chen D, Lam AK (2007) Intrasession and intersession repeatability of the Pentacam system on posterior corneal assessment in the normal human eye. J Cataract Refract Surg 33:448–454. 17321396 10.1016/j.jcrs.2006.11.008

[33] Chen W, McAlinden C, Pesudovs K, Wang Q, Lu F, et al (2012) Scheimpflug-Placido topographer and optical low-coherence reflectometry biometer: repeatability and agreement. J Cataract Refract Surg 38:1626–1632. 22763002 10.1016/j.jcrs.2012.04.031

[34] Mihaltz K, Kovacs I, Takacs A, Nagy ZZ (2009) Evaluation of keratometric, pachymetric, and elevation parameters of keratoconic corneas with pentacam. Cornea 28:976–980. 19724217 10.1097/ICO.0b013e31819e34de

[35] Savini G, Barboni P, Carbonelli M, Hoffer K (2009) Agreement between Pentacam and videokeratography in corneal power assessment. J Refract Surg 25:534–538. 19603621 10.3928/1081597X-20090512-07

[36] Read SA, Collins MJ, Iskander DR, Davis BA (2009) Corneal topography with Scheimpflug imaging and videokeratography: comparative study of normal eyes. J Cataract Refract Surg 35:1072–1081. 19465294 10.1016/j.jcrs.2009.01.020

[37] Huynh SC, Mai TQ, Kifley A, Wang JJ, Rose KA, et al (2006) An evaluation of keratometry in 6-year-old children. Cornea 25:383–387. 16670473 10.1097/01.ico.0000214203.84081.ec

[38] Gatinel D, Haouat M, Hoang-Xuan T (2002) A review of mathematical descriptors of corneal asphericity. J Fr Ophtalmol 25:81–90. 11965125

[39] Read SA, Collins MJ, Carney LG, Franklin RJ (2006) The topography of the central and peripheral cornea. Invest Ophthalmol Vis Sci 47:1404–1415. 16565374 10.1167/iovs.05-1181

[40] McAlinden C, Khadka J, Pesudovs K (2011) A Comprehensive Evaluation of the Precision (Repeatability and Reproducibility) of the Oculus Pentacam HR. Invest Ophthalmol Vis Sci 52:7731–7737. 21810981 10.1167/iovs.10-7093

[41] Delrivo M, Ruisenor Vazquez PR, Galletti JD, Garibotto M, Bonthoux FF, et al (2014) Agreement between placido topography and scheimpflug tomography for corneal astigmatism assessment. J Refract Surg 30:49–53. 24864328 10.3928/1081597X-20131217-06

[42] Muller LJ, Pels E, Vrensen GF (2001) The specific architecture of the anterior stroma accounts for maintenance of corneal curvature. Br J Ophthalmol 85:437–443. 11264134 10.1136/bjo.85.4.437 PMC1723934

